# Supplementary material for: SMN Deficiency Induces an Early Non-Atrophic Myopathy with Alterations in the Contractile and Excitatory Coupling Machinery of Skeletal Myofibers in the SMN∆7 Mouse Model of Spinal Muscular Atrophy
Source: Int J Mol Sci. 2024 Nov 19;25(22):12415. doi: 10.3390/ijms252212415 (PMC11595111; doi:10.3390/ijms252212415)
Supplement: Supplementary file 1 [file ijms-25-12415-s001.zip › ijms-3231221 supplementary figures .pdf]

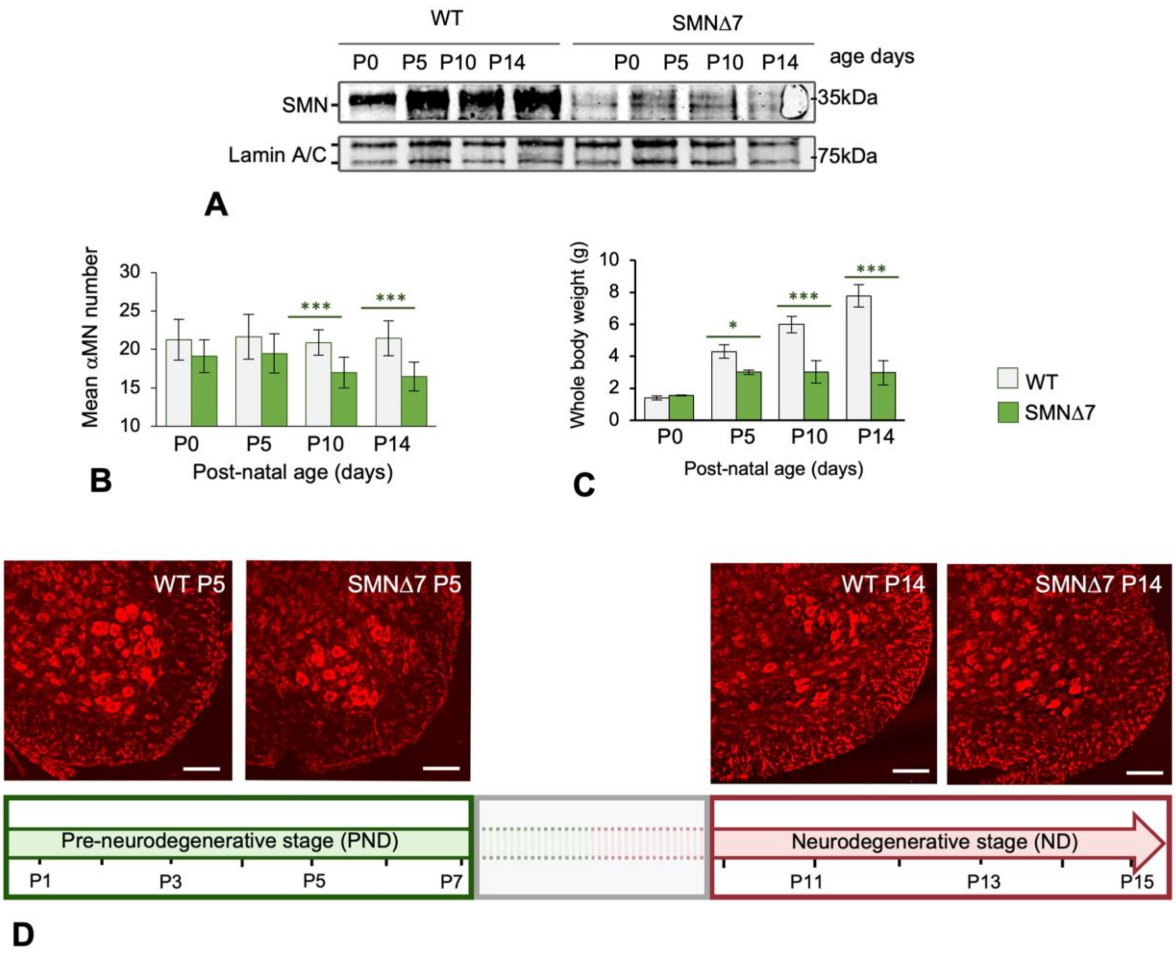

Supplementary figure S1. (A) Western blotting analysis of SMN expression in TA muscle lysates from WT and SMNΔ7 mice at the indicated postnatal ages (P). Lamin A/C was used as load control. Protein expression fold of SMN protein is indicated. **(B)** Quantitative analysis of the mean number of αMNs present in transversal cryosections of spinal cords stained with propidium iodide from WT (n=6) and SMNΔ7 (n=6) mice at the indicated postnatal ages (P). *p* values from WT and SMNΔ7 data comparison were 0.0865, 0.0727, 2.6-04 and 9.8E-11 at P0, P5, P10 and P14, respectively. **(C)** Quantitative analysis of body weight changes in WT (n=12) and SMNΔ7 (n=12) mice at indicated postnatal ages. **(D)** Representative confocal images of transversal cryosections of anterior horns stained with propidium iodide from WT and SMNΔ7 mice at P5 and P14 used for the αMN quantification shown in panel B**.** Schematic illustration of the time-course of pre-neurodegenerative (PND) and neurodegenerative (ND) stages. Scale bar = 200 μm in (D).

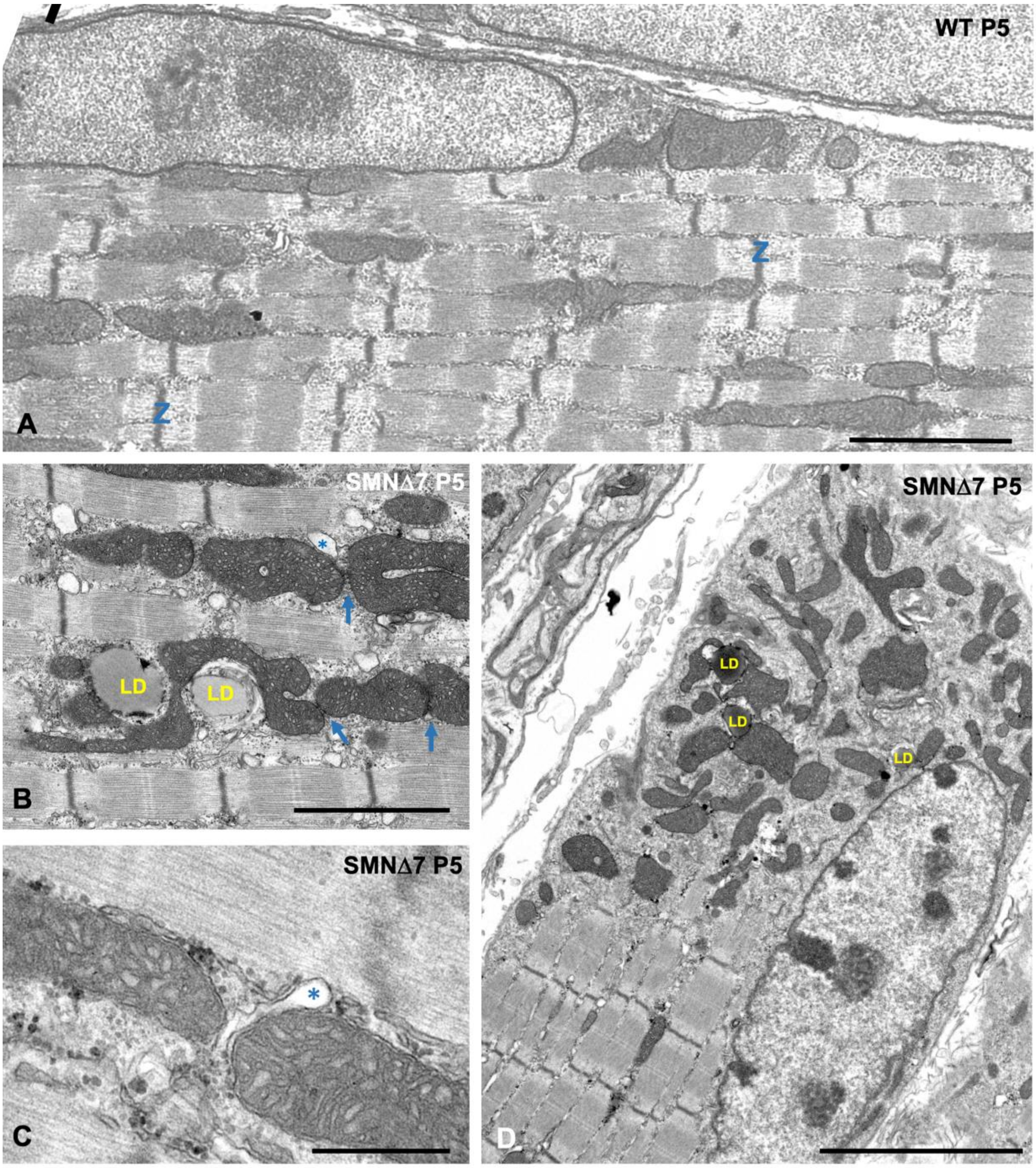

Supplementary figure S2. (A) Electron micrograph showing the ultrastructural phenotype and organization of subsarcolemmal and intermyofibrillar mitochondria in a WT myofiber at P5. Z: Z-discs. **(B-D)** Fine structure of mitochondria in SMNΔ7 myofibers. **(B)** Clusters of intermyofibrillar mitochondria illustrating the direct link between the outer membranes of adjacent mitochondria (arrows) and the interactome of mitochondria with both lipid droplets (LD) and dilated SR cisterns (asterisk). **(C)** High magnification detail of the close interaction between the outer mitochondrial membrane and the membrane of a dilated SR cistern (asterisk). Note the well-preserved ultrastructure of mitochondria. **(D)** Great accumulation of subsarcolemmal mitochondria of variable size and morphology. Note the absence of mitophagy images. LD: lipid droplets. Scale bar = 2 μm (A, B), 200nm (C) and 5 μm (D).
